# Supplementary material for: Clinical NEC prevention practices drive different microbiome profiles and functional responses in the preterm intestine
Source: Nat Commun. 2023 Mar 11;14:1349. doi: 10.1038/s41467-023-36825-1 (PMC10008552; doi:10.1038/s41467-023-36825-1)
Supplement: Supplementary file 1 — Supplementary Information [file 41467_2023_36825_MOESM1_ESM.pdf]

Supplementary Information

Clinical NEC prevention practices drive different microbiome profiles and functional responses in the preterm intestine

Charlotte J Neumann[1], Alexander Mahnert[1], Christina Kumpitsch[1], Raymond Kiu[2], Matthew J Dalby[2], Magdalena Kujawska[3], Tobias Madl[4,5], Stefan Kurath-Koller[6], Berndt Urlesberger[7,8], Bernhard Resch[7,8*], Lindsay J Hall[2,3,9], Christine Moissl-Eichinger[1,5*+]

ASV-tables and metabolomic, as well as metabolomic data are shared via Github (https://github.com/CharlotteJNeumann/preterm_shared).

This Supplementary Information contains:

- Suppl. Fig. 1: Feeding history of all infants
- Suppl. Fig. 2: Network analysis
- Suppl. Fig. 3: Correlation of *Staphylococcus* and *Staphylococcus* phages
- Suppl. Fig. 4: Normalized integrals of several metabolites
- Suppl. Fig. 5: Complete heat map of correlation of metabolites and key-taxa
- Suppl. Fig. 6: Flowchart of the study design

- Suppl. Table 1: PERMANOVA analysis
- Suppl. Table 2: Time point of stool sample collections
- Suppl. Table 3a: Alignment parameters of amplicon reads aligned with probiotic reference 16S rRNA genes
- Suppl. Table 3b: FastANI score for MAGs classified as *Bifidobacterium* in Klagenfurt samples
- Suppl. Table 4: Distribution of overall reads

Suppl. Fig 1: Feeding history of all infants per time point

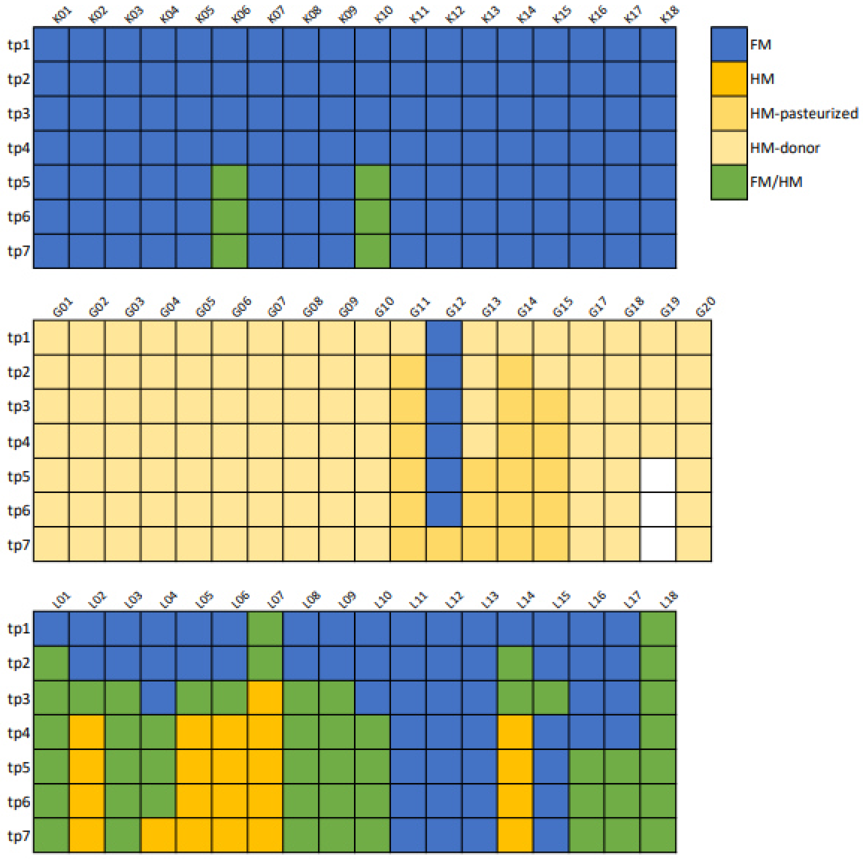

Suppl. Fig. 2 Network analysis of selected genera from each methanogens, ascomycota/Basidiomycota, phages and bacteria. Nodes represent taxa and edges positive and negative co-occurences according to SparCC R values.

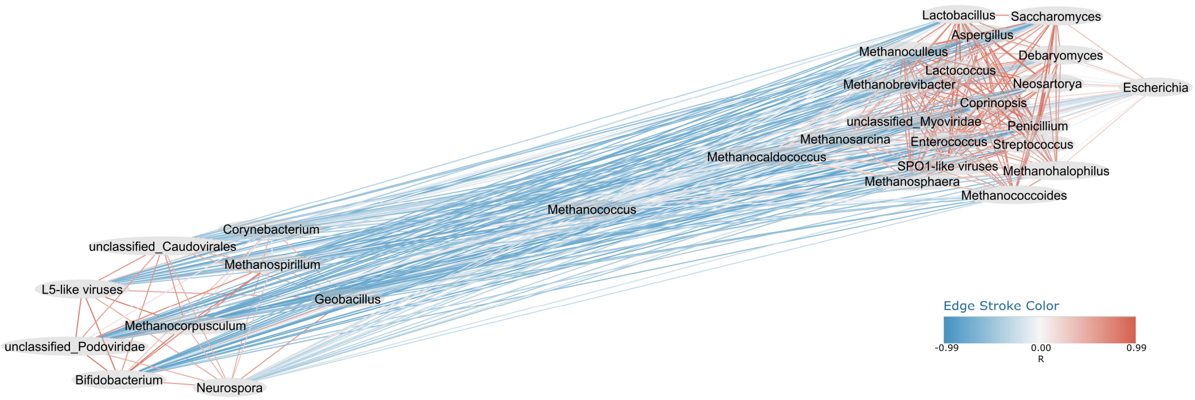

Suppl. Fig. 3: Spearman correlation of Staphylococcus with Staphylococcus phages

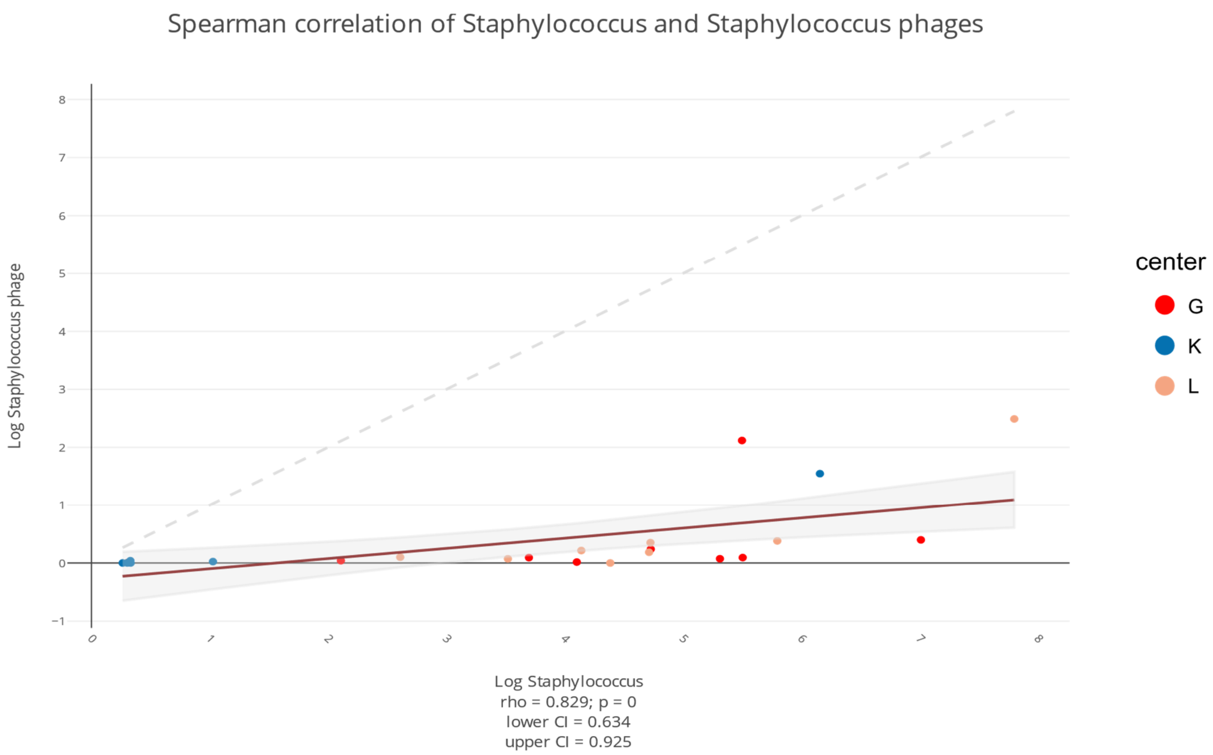

Suppl. Fig 4: Normalized integrals of several metabolites over time (tp1, tp3, tp7) and centers (G, K, L) n= 151, Kruskal-Wallis corrected by Bonferroni; A) acetic acid, B) formic Acid, C) valeric acid, D) butyric acid, E) propionic acid, F) Glucose, and G) Fructose; For boxplots, the upper, middle and lower horizontal lines of the box represent the upper, median and lower quartile; their whiskers depict the smallest or largest values within 1.5-fold of the interquartile range. Significance levels are indicated with asterisks for q < 0.001 (***), q < 0.01 (**), q < 0.05 (*). Centers are abbreviated by G (Graz), L (Leoben) and K (Klagenfurt).

Acetic acid: K1:K3 q = 1.000; K1:K7 q = 1.000.; K3:K7 q = 1.000; G1:G3 q = 1.000.; G1:G7 q = 0.298; G3:G7 q = 1.000; L1:L3 q = = 1.000; L1:L7 q < 0.001.; L3:L7 q = 0.001; G1:K1 q = 1.000; G3:K3 q = 1.000; G7:K7 q = 1.000; L1:K1 q = 1.000; L3:K3 q = 1.000; L7:K7 q = 1.000; G1:L1 q = 1.000; G3:L3 q =1.000; G7:L7 q = 1.000;

Formic acid: K1:K3 q = 1.000; K1:K7 q = 0.977; K3:K7 q = 0.745; G1:G3 q = 1.000; G1:G7 q = 0.046; G3:G7 q = 1.000; L1:L3 q = 1.000.; L1:L7 q = 1.000; L3:L7 q = 1.000; G1:K1 q = 1.000; G3:K3 q = 1.000; G7:K7 q = 1.000; L1:K1 q = 1.000; L3:K3 q = 1.000; L7:K7 q = 1.000; G1:L1 q = 1.000; G3:L3 q = 1.000; G7:L7 q = 1.000;

Valeric acid: K1:K3 q = 1.000; K1:K7 q = 0.001; K3:K7 q = 0.004; G1:G3 q = 0.032; G1:G7 q = 0.093; G3:G7 q = 1.000; L1:L3 q = 1.000; L1:L7 q = 0.903; L3:L7 q = 0.288; G1:K1 q = 1.000; G3:K3 q = 0.518; G7:K7 q = 1.000; L1:K1 q = 1.000; L3:K3 q = 1.000 ; L7:K7 q = 1.000; G1:L1 q = 1.000; G3:L3 q = 0.218; G7:L7 q = 1.000;

Butyric acid: K1:K3 q = 1.000; K1:K7 q = 1.000; K3:K7 q = 1.000; G1:G3 q = 1.000; G1:G7 q = 1.000; G3:G7 q = 0.158; L1:L3 q = 0.095; L1:L7 q = 0.054; L3:L7 q = 1.000; G1:K1 q = 1.000; G3:K3 q = 0.027; G7:K7 q = 1.000; L1:K1 q = 1.000; L3:K3 q = 0.039 ; L7:K7 q = 1.000; G1:L1 q = 1.000; G3:L3 q = 1.000; G7:L7 q = 1.000;

Propionic acid: K1:K3 q = 0.019; K1:K7 q = 1.000; K3:K7 q = 0.226; G1:G3 q < 0.001; G1:G7 q = 1.000; G3:G7 q = 0.071; L1:L3 q < 0.001; L1:L7 q = 0.211; L3:L7 q = 0.918; G1:K1 q = 1.000; G3:K3 q = 1.000; G7:K7 q = 1.000; L1:K1 q = 1.000; L3:K3 q = 1.000; L7:K7 q = 1.000; G1:L1 q = 1.000; G3:L3 q = 1.000; G7:L7 q = 1.000;

Glucose: K1:K3 q = 1.000; K1:K7 q = 1.000; K3:K7 q = 1.000; G1:G3 q < 0.001; G1:G7 q < 0.001; G3:G7 q = 1.000; L1:L3 q < 0.001; L1:L7 q < 0.001; L3:L7 q = 1.000; G1:K1 q = 1.000; G3:K3 q = 0.001; G7:K7 q = 0.207; L1:K1 q = 1.000; L3:K3 q < 0.001; L7:K7 q = 0.185; G1:L1 q = 1.000; G3:L3 q = 1.000; G7:L7 q = 1.000;

Fructose: K1:K3 q = 1.000; K1:K7 q = 0.338; K3:K7 q = 0.008; G1:G3 q = 0.001; G1:G7 q < 0.001; G3:G7 q = 1.000; L1:L3 q = 0.072; L1:L7 q = 0.001; L3:L7 q = 1.000; G1:K1 q = 1.000; G3:K3 q < 0.001; G7:K7 q = 1.000; L1:K1 q = 1.000; L3:K3 q < 0.001 ; L7:K7 q = 1.000; G1:L1 q = 1.000; G3:L3 q = 1.000; G7:L7 q = 1.000;

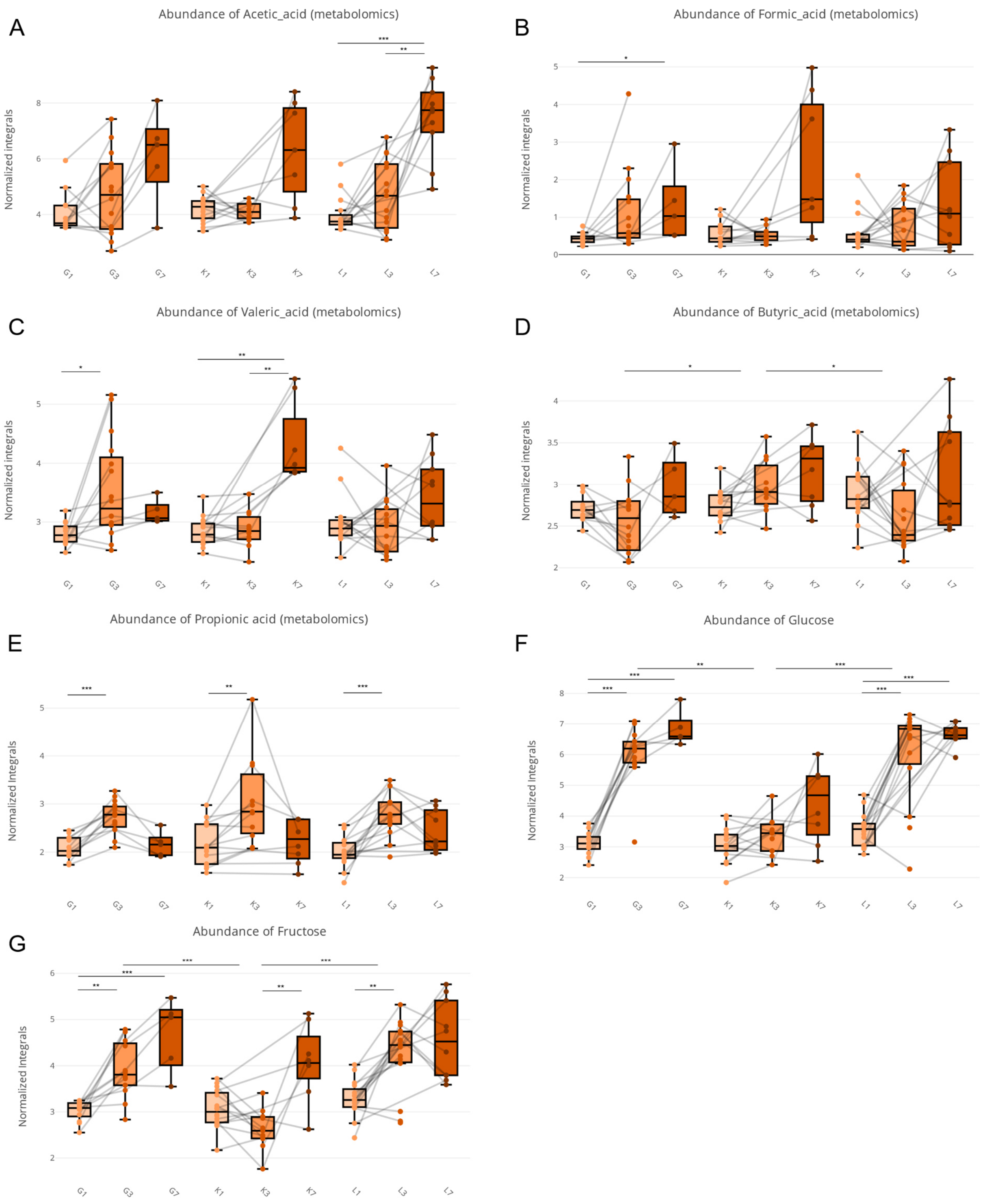

A
Abundance of Acetic_acid (metabolomics)
Normalized integrals
B
Abundance of Formic_acid (metabolomics)
Normalized integrals
C
Abundance of Valeric_acid (metabolomics)
Normalized integrals
D
Abundance of Butyric_acid (metabolomics)
Normalized integrals
E
Abundance of Propionic acid (metabolomics)
Normalized Integrals
F
Abundance of Glucose
Normalized Integrals
G
Abundance of Fructose
Normalized Integrals
G1
G3
G7
K1
K3
K7
L1
L3
L7

Suppl. Fig. 5: Complete heat map of correlation of metabolites and key-taxa of the three centers. Significance levels are indicated with asterisks for q < 0.001 (***), q < 0.01 (**), q < 0.05 (*) by Pearson corrected for multiple testing by Benjamini-Hochberg.

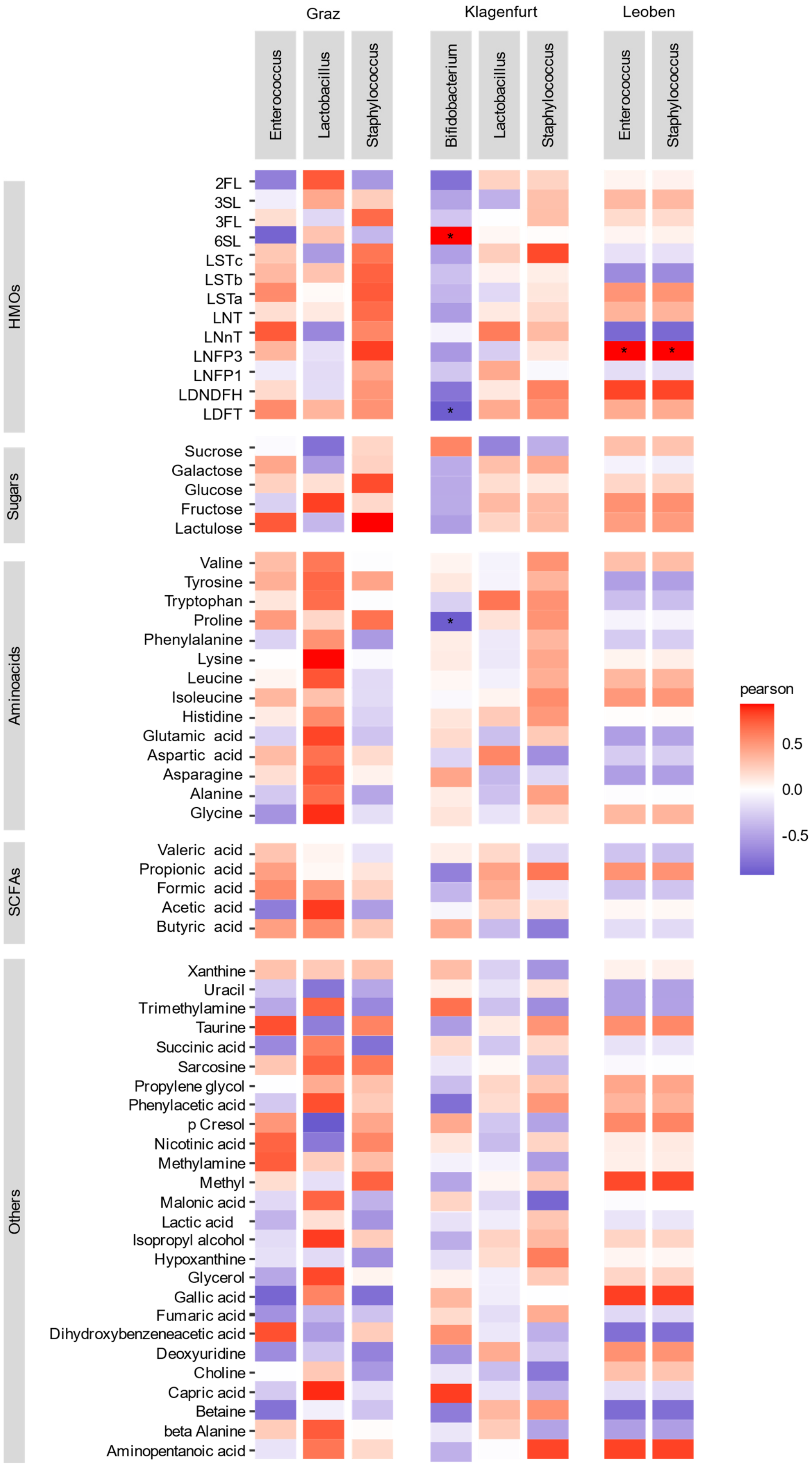

Graz
Klagenfurt
Leoben
Enterococcus
Lactobacillus
Staphylococcus
Bifidobacterium
Lactobacillus
Staphylococcus
Enterococcus
Staphylococcus
HMOs
2FL
3SL
3FL
6SL
LSTc
LSTb
LSTa
LNT
LNnT
LNFP3
LNFP1
LDNDFH
LDFT
Sugars
Sucrose
Galactose
Glucose
Fructose
Lactulose
Aminoacids
Valine
Tyrosine
Tryptophan
Proline
Phenylalanine
Lysine
Leucine
Isoleucine
Histidine
Glutamic acid
Aspartic acid
Asparagine
Alanine
Glycine
SCFAs
Valeric acid
Propionic acid
Formic acid
Acetic acid
Butyric acid
Others
Xanthine
Uracil
Trimethylamine
Taurine
Succinic acid
Sarcosine
Propylene glycol
Phenylacetic acid
p Cresol
Nicotinic acid
Methylamine
Methyl
Malonic acid
Lactic acid
Isopropyl alcohol
Hypoxanthine
Glycerol
Gallic acid
Fumaric acid
Dihydroxybenzeneacetic acid
Deoxyuridine
Choline
Capric acid
Betaine
beta Alanine
Aminopentanoic acid
pearson
0.5
0.0
-0.5

Suppl. Fig. 6: Flowchart of the Study design

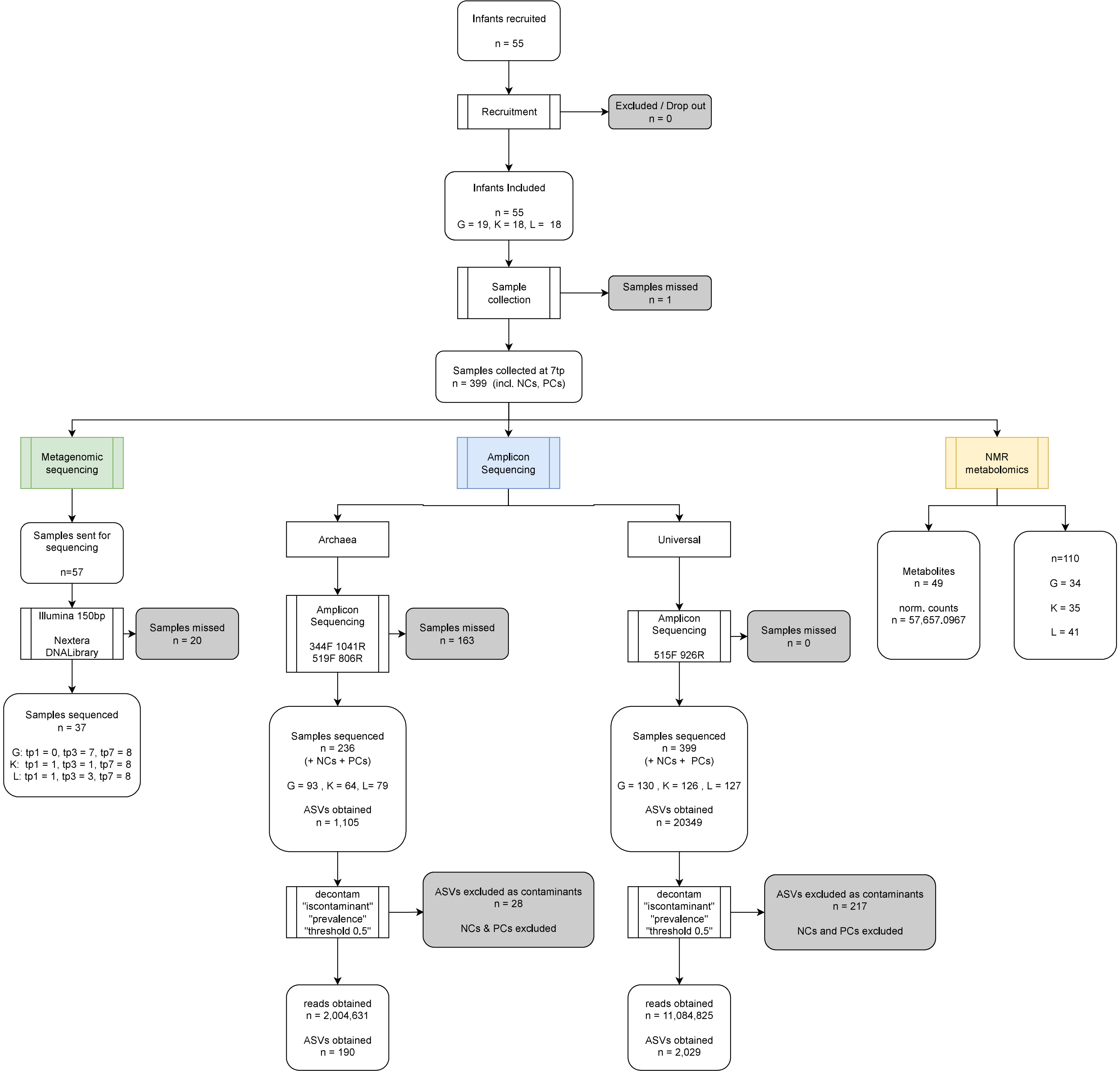
Infants recruited
n = 55
Recruitment
Excluded / Drop out
n = 0
Infants Included
n = 55
G = 19, K = 18, L = 18
Sample collection
Samples missed
n = 1
Samples collected at 7tp
n = 399 (incl. NCs, PCs)
Metagenomic sequencing
Amplicon Sequencing
NMR metabolomics
Samples sent for sequencing
n=57
Illumina 150bp
Nextera DNALibrary
Samples missed
n = 20
Samples sequenced
n = 37
G: tp1 = 0, tp3 = 7, tp7 = 8
K: tp1 = 1, tp3 = 1, tp7 = 8
L: tp1 = 1, tp3 = 3, tp7 = 8
Archaea
Amplicon Sequencing
344F 1041R
519F 806R
Samples missed
n = 163
Samples sequenced
n = 236
(+ NCs + PCs)
G = 93 , K = 64, L= 79
ASVs obtained
n = 1,105
decontam
"iscontaminant"
"prevalence"
"threshold 0.5"
ASVs excluded as contaminants
n = 28
NCs & PCs excluded
reads obtained
n = 2,004,631
ASVs obtained
n = 190
Universal
Amplicon Sequencing
515F 926R
Samples missed
n = 0
Samples sequenced
n = 399
(+ NCs + PCs)
G = 130 , K = 126 , L = 127
ASVs obtained
n = 20349
decontam
"iscontaminant"
"prevalence"
"threshold 0.5"
ASVs excluded as contaminants
n = 217
NCs and PCs excluded
reads obtained
n = 11,084,825
ASVs obtained
n = 2,029
Metabolites
n = 49
norm. counts
n = 57,657.0967
n=110
G = 34
K = 35
L = 41

Suppl. Table 1: PERMANOVA (p-value: 0.001 for all tests), amplicon data

**Variable tested**	**R^2, all time points**	**R^2, tp 3**	**R^2, tp 7**
Bifidobacterium administration	0.2083545	0.1258109	0.5715891
Lactobacillus administration	0.04733006	0.0631141	0.139399
Hospital	0.2622558	0.2618448	0.6762656
Nutrition[1]	0.1176184	0.1611184	0.3566395
Nutrition[1]_*bifidobacterium*[2]	0.2512166	0.3003471	0.6545865
Nutrition[2]_*bifidobacterium*[2]-Gentamycin[3]	0.270972	0.3429452	0.660657

[1] (FM, MM, mixed)
[2] *Bifidobacterium* administration: yes/no
[3] Gentamycin administration: yes/no

Suppl. Table 2: timepoint of stool sample collections

Time Points	Minimum	Maximum	Mean
tp1	1	3	1.56
tp2	3	6	3.75
tp3	5	8	5.82
tp4	7	15	8.04
tp5	9	13	10.06
tp6	11	15	12.15
tp7	13	21	14.50

Suppl. Table 3a: Alignment parameters of amplicon reads aligned with probiotic reference 16S rRNA genes

Center	Species	Max. score	Total score	Query covery	E value	% identity	Acc. Length
K	*L. acidophilus*	719	719	100%	0	100.00%	1559
G	*L. rhamnosus*	691	691	100%	0	100.00%	1567

Suppl. Table 3b: FastANI score for MAGs classified as Bifidobacterium in Klagenfurt samples

MAGs	classification	FastANI
K08-7.MAG.001.fasta_assembly	g__Bifidobacterium;s__Bifidobacterium infantis	100
K11-7.MAG.001.fasta_assembly	g__Bifidobacterium;s__Bifidobacterium infantis	100
K13-7.MAG.001.fasta_assembly	g__Bifidobacterium;s__Bifidobacterium infantis	100
K14-7.MAG.001.fasta_assembly	g__Bifidobacterium;s__Bifidobacterium infantis	99.99
K15-7.MAG.001.fasta_assembly	g__Bifidobacterium;s__Bifidobacterium infantis	100
K16-7.MAG.002.fasta_assembly	g__Bifidobacterium;s__Bifidobacterium infantis	99.97
K17-7.MAG.001.fasta_assembly	g__Bifidobacterium;s__Bifidobacterium infantis	99.99

Suppl. Table 4: Distribution of overall reads (56,660,357) into Archaea, Ascomycetes-Basidiomycetes, Viruses and Bacteria as well as in their highest abundant subgroups. Distribution is given in overall reads and percentages and each for the three centers.

	**Archaea**	**Fungi: Asco-Basidio**		**Viruses**	**Bacteria**					
**all**	24,081 (0.04%)	53,192 (0.09%)		83,670 (0.15%)	56,446,924 (99.62%)					
**G**	8,906 (36.98%)	11,522 (21.66%)		20,860 (24.93%)	15,677,124 (27.77%)					
**K**	6,937 (28.81%)	14,918 (28.05%)		30,894 (36.92%)	20,204,643 (35.79%)					
**L**	8,238 (34.21%)	26,752 (50.29%)		31,916 (38.15%)	20,565,157 (36.43%)					
	Methano-gens	Asco-mycota	Basidio-mycota	Phages						
**all**	15,040 (62.46%)	13,662 (25.68%)	3,9530 (74.32%)	83,670 (100%)						
**G**	5,482 (36.45%)	5,632 (41.22%)	5,890 (14.90%)	20,860 (24.93%)						
**K**	4,813 (32.00%)	1,298 (9.50%)	13,620 (34.45%)	30,894 (36.92%)						
**L**	4,745 (31.55%)	6,732 (49.28%)	20,020 (50.65%)	31,916 (38.15%)						
	Methano-brevibacter				*Enterococcus*	*Bifidobacterium*	*Geobacillus*	*Lactobacillus*	*Escherichia*	*Staphylococcus*
**all**	2,193 (14.58%)				28,334,208 (50.20%)	16,144,186 (28.60%)	289,736 (0.51%)	1,501,052 (2.66%)	1,187,293 (2.10%)	2,414,309 (4.28%)
**G**	943 (43.00%)				11,089,852 (39.14%)	11,506 (0.07%)	38,973 (13.45%)	853,834 (56.88%)	36,431 (3.07%)	827,634 (34.28%)
**K**	99 (4.51%)				1,506,496 (5.32%)	16,115,145 (99.82%)	202,224 (69.80%)	74,547 (4.97%)	1,145,599 (96.49%)	258,229 (10.70%)
**L**	1,151 (52.49%)				15,737,860 (55.54%)	17,535 (0.11%)	48,539 (16.75%)	572,671 (38.15%)	5,263 (0.44%)	1,328,446 (55.02%)
