# Supplementary material for: Clinical NEC prevention practices drive different microbiome profiles and functional responses in the preterm intestine
Source: Nat Commun. 2023 Mar 11;14:1349. doi: 10.1038/s41467-023-36825-1 (PMC10008552; doi:10.1038/s41467-023-36825-1)
Supplement: Supplementary file 3 — Reporting Summary [file 41467_2023_36825_MOESM3_ESM.pdf]

nature portfolio

Corresponding author(s): Christine Moissl-Eichinger

Last updated by author(s): 06_11_2022

Reporting Summary

Nature Portfolio wishes to improve the reproducibility of the work that we publish. This form provides structure for consistency and transparency in reporting. For further information on Nature Portfolio policies, see our Editorial Policies and the Editorial Policy Checklist.

Statistics

For all statistical analyses, confirm that the following items are present in the figure legend, table legend, main text, or Methods section.

n/a	Confirmed	
☐	☒	The exact sample size (*n*) for each experimental group/condition, given as a discrete number and unit of measurement
☒	☐	A statement on whether measurements were taken from distinct samples or whether the same sample was measured repeatedly
☐	☒	The statistical test(s) used AND whether they are one- or two-sided<br>*Only common tests should be described solely by name; describe more complex techniques in the Methods section.*
☐	☒	A description of all covariates tested
☐	☒	A description of any assumptions or corrections, such as tests of normality and adjustment for multiple comparisons
☐	☒	A full description of the statistical parameters including central tendency (e.g. means) or other basic estimates (e.g. regression coefficient) AND variation (e.g. standard deviation) or associated estimates of uncertainty (e.g. confidence intervals)
☐	☒	For null hypothesis testing, the test statistic (e.g. *F*, *t*, *r*) with confidence intervals, effect sizes, degrees of freedom and *P* value noted<br>*Give P values as exact values whenever suitable.*
☒	☐	For Bayesian analysis, information on the choice of priors and Markov chain Monte Carlo settings
☒	☐	For hierarchical and complex designs, identification of the appropriate level for tests and full reporting of outcomes
☒	☐	Estimates of effect sizes (e.g. Cohen's *d*, Pearson's *r*), indicating how they were calculated

Our web collection on statistics for biologists contains articles on many of the points above.

Software and code

Policy information about availability of computer code

Data collection	All information provided in Materials and Method section
Data analysis	All details are provided in Materials and Method section.

For manuscripts utilizing custom algorithms or software that are central to the research but not yet described in published literature, software must be made available to editors and reviewers. We strongly encourage code deposition in a community repository (e.g. GitHub). See the Nature Portfolio guidelines for submitting code & software for further information.

Data

Policy information about availability of data

All manuscripts must include a data availability statement. This statement should provide the following information, where applicable:

- Accession codes, unique identifiers, or web links for publicly available datasets
- A description of any restrictions on data availability
- For clinical datasets or third party data, please ensure that the statement adheres to our policy

The datasets generated during and/or analysed during the current study are available in the github repository https://github.com/CharlotteJNeumann/preterm_shared

Human research participants

Policy information about studies involving human research participants and Sex and Gender in Research.

Reporting on sex and gender	The sex and gender of the infants did not influence the recruitment. The sex was included into analyses as possible covariates but did not give insight into any statistically significant or nameworthy differences between groups.
Population characteristics	Covariate-relevant population characteristics were birth weight, survival of the first three weeks of life, genetic diseases, syndroms or congenital anomalies as well as meconium ileus. Further details are given in the manuscript itself and previous publications (Kurath-Koller et al., 2017 and 2020).
Recruitment	Inclusion criteria included a birth weight <1500 g, treatment at the local NICUs between October 2015 and March 2017, and survival of the first three weeks of life. Exclusion criteria included birth weight ≥1500 g, death within the first three weeks of life, genetic diseases, syndromes or congenital anomalies as well as meconium ileus. All infants were recruited after receiving parents´ written informed consent. Further details are given in the manuscript itself and previous publications (Kurath-Koller et al., 2017 and 2020).
Ethics oversight	The local ethic committees approved the study protocol (number 27-366 ex14/15)

Note that full information on the approval of the study protocol must also be provided in the manuscript.

Field-specific reporting

Please select the one below that is the best fit for your research. If you are not sure, read the appropriate sections before making your selection.

☒ Life sciences ☐ Behavioural & social sciences ☐ Ecological, evolutionary & environmental sciences

For a reference copy of the document with all sections, see nature.com/documents/nr-reporting-summary-flat.pdf

Life sciences study design

All studies must disclose on these points even when the disclosure is negative.

Sample size	A study group was recruited that is homogeneous among the centers. No statistical analysis was performed to detect sample size.
Data exclusions	See provided STORM chart.
Replication	N.a.
Randomization	N.a.
Blinding	N.a.

Reporting for specific materials, systems and methods

We require information from authors about some types of materials, experimental systems and methods used in many studies. Here, indicate whether each material, system or method listed is relevant to your study. If you are not sure if a list item applies to your research, read the appropriate section before selecting a response.

Materials & experimental systems

n/a	Involved in the study	
☒	☐	Antibodies
☒	☐	Eukaryotic cell lines
☒	☐	Palaeontology and archaeology
☒	☐	Animals and other organisms
☐	☒	Clinical data
☒	☐	Dual use research of concern

Methods

n/a	Involved in the study	
☒	☐	ChIP-seq
☒	☐	Flow cytometry
☒	☐	MRI-based neuroimaging

Clinical data

Policy information about clinical studies

All manuscripts should comply with the ICMJE guidelines for publication of clinical research and a completed CONSORT checklist must be included with all submissions.

Clinical trial registration	The study has been registered with the German Registry for Clinical Trials (registry ID DRKS00009290)
Study protocol	Kurath-Koller, S. et al. Changes of intestinal microbiota composition and diversity in very low birth weight infants related to strategies of NEC prophylaxis: protocol for an observational multicentre pilot study. Pilot Feasibility Stud. 3, 52 (2017).
Data collection	Between October 2015 and March 2017, stool samples were collected from preterm infants at those three centers. Inclusion criteria were birth weight < 1,500 g and survival in the first three weeks of life. In case of genetic diseases, syndromes or congenital anomalies or meconium ileus, infants were excluded from the study. A total of 54 infants were included in the study. The infants' stool samples were collected every other day from meconium for the first two weeks of life, with each infant providing stool samples at seven time points (timepoints of samples were slightly variable due to the varying availability of fecal samples; average sampling time points were tp1: within day 1-3; tp2: day 4; tp3: day 6; tp4: day 8; tp5: day 10; tp6: day12; tp7: day 15). A total of 383 samples and 16 negative controls were collected.<br>several clinical data were collected, such as: sex, birth weight (g), gestational age, APGAR 1, APGAR 5, APGAR 10, Ventilation (d), Supplied oxygen, birthmode, length of hospital stay (d), maternal age (years), multiples, UApH, breast milk feeding, EOS, LOS, I/PVH, RDS, ROP, incidence of NEC
Outcomes	Primary outcomes are changes of microbiome compositions. Secondary outcomes are metabolomic data.<br>See M&M
