# Supplementary material for: Clinical NEC prevention practices drive different microbiome profiles and functional responses in the preterm intestine
Source: Nat Commun. 2023 Mar 11;14:1349. doi: 10.1038/s41467-023-36825-1 (PMC10008552; doi:10.1038/s41467-023-36825-1)
Supplement: Supplementary file 4 — Source Data [file 41467_2023_36825_MOESM4_ESM.pdf]

	Link to file in https://github.com/CharlotteJNeumann/preterm_shared	comment
Figure 1		
a	preterm_shared/ASV_table_archaea/ASV_table.txt	Microbiome Explorer, CSS normalization
	preterm_shared/ASV_table_archaea/metadata.txt	
	preterm_shared/ASV_table_archaea/taxonomy.txt	
b	preterm_shared/Metagenomics/taxonomy/data_table_metagenomics_methanogens.txt	Microbiome Explorer, CSS normalization
	preterm_shared/Metagenomics/taxonomy/metadata.txt	
	preterm_shared/Metagenomics/taxonomy/taxonomy_archaea.txt	
c	preterm_shared/Metagenomics/taxonomy/featureCounts_metag_methanogens_genus_CSS_prop.csv	data
	preterm_shared/Metagenomics/taxonomy/DESeq2_metagenomics_methanogens_genus.txt	DSeq2-values
d	preterm_shared/Metagenomics/taxonomy/featureCounts_metagenomics_ITS_genus_CSS_prop.csv	data
	preterm_shared/Metagenomics/taxonomy/DESeq2_metagenomics_ITS_genus.txt	DSeq2-values
e	preterm_shared/Metagenomics/taxonomy/data_table_metagenomics_ITS.txt	Microbiome Explorer, CSS normalization
	preterm_shared/Metagenomics/taxonomy/metadata.txt	
	preterm_shared/Metagenomics/taxonomy/taxonomy_ITS.txt	
f	preterm_shared/Metagenomics/taxonomy/data_table_viruses.txt	Microbiome Explorer, CSS normalization
	preterm_shared/Metagenomics/taxonomy/metadata.txt	
	preterm_shared/Metagenomics/taxonomy/data_table_viruses.txt	
Figure 2		
a	preterm_shared/ASV_table_universal/featureCounts_amplicon_bacteria_genus_tp7_CSS_prop.csv	data
	preterm_shared/ASV_table_universal/DESeq2_amplicon_universal_genus.txt	DSeq2-values
b	preterm_shared/ASV_table_universal/ASV_table.txt	Microbiome Explorer, CSS normalization
	preterm_shared/ASV_table_universal/metadata.txt	
	preterm_shared/ASV_table_universal/taxonomy.txt	
c	preterm_shared/Metagenomics/Krona-Lactobacillus.xlsm	data
d	preterm_shared/ASV_table_universal/featureCounts_metagenomics_bacteria_species_CSS_normalized	data
	preterm_shared/ASV_table_universal/2d_DSeq2_metagenomics_bacteria_species	DSeq2-values
e	preterm_shared/R_script_BioEnV	script and data
Figure 3		
a	preterm_shared/Metagenomics/contigs/iRep.txt	data file
b	preterm_shared/Metagenomics/taxonomy/data_table_NEC.txt	Microbiome Explorer, CSS normalization
	preterm_shared/Metagenomics/taxonomy/metadata.txt	
	preterm_shared/Metagenomics/taxonomy/taxonomy_NEC.txt	
c	preterm_shared/Metagenomics/function/data_table_function.txt	Microbiome Explorer, CSS normalization
	preterm_shared/Metagenomics/function/metadata.txt	
d	preterm_shared/Metagenomics/function/featureCounts_functions_order_CSS.csv	data file 1
	preterm_shared/Metagenomics/function/featureCounts_functions_class_CSS.csv	data file 2
	preterm_shared/Metagenomics/function/DSeq2_functions_order.txt	DSeq2-values 1
	preterm_shared/Metagenomics/function/DSeq2_functions_class.txt	DSeq2-values 2
Figure 4		
a	preterm_shared/Metagenomics/data_table_sugars.txt	data file
b	preterm_shared/Metabolomics/data_table_HMOs.txt	data file
c	preterm_shared/Metabolomics/data_table_metabolites.txt	data file
d	preterm_shared/Metagenomics/abricate/HMOs.txt	data file
	preterm_shared/Metagenomics/contigs	sequences of MAGs
Figure 5		
a	preterm_shared/R_script_heatmap_corr_metabolites_genera	all files in the folder
b	preterm_shared/heatmap_antibiotic_resistances/data_table_antibiotic_resistances.txt	data file
	preterm_shared/heatmap_antibiotic_resistances/metadata.txt	metadata
c	preterm_shared/Metagenomics/resfinder.txt	data file
	preterm_shared/Metagenomics/contigs	sequences of MAGs
d	preterm_shared/Metagenomics/vfdb.txt	data file
	preterm_shared/Metagenomics/contigs	sequences of MAGs
